# 

**Published:** 1899-04-22

**Authors:** 


**Notices of Births, Deaths, and Marriages** are inserted in this column at a charge of 2s. 6d. for an announcement not exceeding four lines.

### NOTICE TO CORRESPONDENTS.

All MS., Letters, Books for Review, and other matters intended for the Editor should be addressed The Editor, "The Hospital," The Hospital Building, 28 & 29, Southampton Street, Strand, W.C.

The Editor cannot undertake to return rejected MS., even when accompanied by stamped directed envelope.

**Notice to Advertisers and Subscribers.**

All Advertisements, Orders for Copies of The Hospital, and Business Communications should be addressed to **THE MANAGER (Not to the Editor)**, The Hospital, The Hospital Building, 28 & 29, Southampton Street, Strand, London, W.C.

The Cost of Subscription, post free, is as follows:—Per week, 2½d.; per quarter, 2s. 8d.; per half-year, 5s. 3d.; per annum, 10s. 6d. Foreign:—Per annum, 15s. 2d., payable, in all cases, in advance.

**NOTICE.—Vol. XXV. of "The Hospital" (October, 1898, to March, 1899), in handsome cloth binding, will shortly be on sale at the Office of this Journal, price 7s. 6d. Cases for binding the Numbers contained in this Volume 1s. 6d. Index to Vol. XXV., 2½d., post free.**

**The Monthly Part for May will be ready May 1st. Price 6d., by post 8d.**

### BOOKS RECEIVED.

Scientific Press.
"Burdett's Official Nursing Directory, 1899."

John Lane.
"Professor Hieronimus." Translated from the Danish of Amalie Skram, by Alice Stronach and G. B. Jacobi.

Dawburn and Ward.
"The Natural Waters of Harrogate." By Francis William Smith, M.D.

Henry J. Glaisher.
"Consumption and its Modern Treatment." By George Herschell, M.D., Lond.

Egerton Smith and Co.
"The Construction of Hospitals for Consumption and other Infectious Diseases." By John W. Hayward, M.D.

E. J. Sproston.
"The Relief of Suffering in Labour." By W. E. Fothergill, M.D.

E. Gould.
"Viscum Album, the Common Mistletoe." By George Black, M.B. Edin.

H. and W. Brown.
"The Use of Tuberculin for Lessening the Prevalence of Tuberculosis." By Harold Scurfield, M.D.

Builders' Journal and Architectural Record.
"Ventilation." By Dr. John W. Hayward.

Washington: Government Printing Office.
"Report of the United States Fish Commission." By Barton Warren Evermann, Ph.D.

Richard Brown.
"Report of the Medical Officer of Health for Bridlington." By William A. Wetwan, M.R.C.S.

